# Exposing a Motherhood Penalty in Sport: A Feminist Narrative Inquiry of Media Stories of Canadian Athlete Mothers’ Journeys to the 2020 Tokyo Games

**DOI:** 10.1177/21674795231187916

**Published:** 2023-07-11

**Authors:** Kerry R. McGannon, Shaantanu Kulkarni, Willa Hladun, Andrea Bundon, Ann Pegoraro

**Affiliations:** 1 School of Kinesiology and Health Sciences, 7728 Laurentian University, Sudbury, ON, Canada; 2 School of Kinesiology, 70435 University of British Columbia, Vancouver, BC, Canada; 3 Gordon S. Lang School of Business and Economics, 3653 University of Guelph, Guelph, ON, Canada

**Keywords:** motherhood, sport, narrative analysis, feminism

## Abstract

Coinciding with athlete mothers’ stories gaining media visibility, sport media researchers are studying media discourses to learn more about socially constructed motherhood and sport. The present study extends media research on elite athlete mothers, by using feminist narrative inquiry to interrogate discrimination meanings in sport. North American sport media stories were collected on Canadian athletes’ (i.e., boxer Mandy Bujold, basketball player Kim Gaucher) journeys to the 2020 Tokyo Games after being discriminated against due to their motherhood status. Thematic narrative analysis of 103 stories identified three narrative motifs (i.e., recurring concepts) in stories linked to discrimination meanings: last shots, forced to choose, and more than us. The first two motifs are discussed in relation to a motherhood penalty narrative linked to sexism and discrimination. The more than us motif is discussed in relation to the resolution to compete for both athletes, linked to maternal activism and social change. All three motifs exposed and challenged maternal discrimination in sport, using ‘feminist consciousness’ linked to a neoliberal feminist status quo. These findings show the pedagogical potential of media stories for athlete maternity rights awareness and structural change, while highlighting a need for intersectional feminist reform regarding athlete parents and post-pandemic recovery.

Coinciding with athlete mothers’ stories gaining media visibility, sport media researchers are studying media discourses to learn more about socially constructed motherhood and sport. The present study extends media research on elite athlete mothers, by using feminist narrative inquiry to interrogate discrimination meanings in sport. North American sport media stories were collected on Canadian athletes’ (i.e., boxer Mandy Bujold, basketball player Kim Gaucher) journeys to the 2020 Tokyo Games after being discriminated against due to their motherhood status. Thematic narrative analysis of 103 stories identified three *narrative motifs* (i.e., recurring concepts) in stories linked to discrimination meanings: *last shots*, *forced to choose*, *and more than us*. The first two *motifs* are discussed in relation to a motherhood penalty narrative linked to sexism and discrimination. The *more than us motif* is discussed in relation to the resolution to compete for both athletes, linked to maternal activism and social change. All three *motifs* exposed and challenged maternal discrimination in sport, using ‘feminist consciousness’ linked to a neoliberal feminist status quo. These findings show the pedagogical potential of media stories for athlete maternity rights awareness and structural change, while highlighting a need for intersectional feminist reform regarding athlete parents and post-pandemic recovery.

Although motherhood is historically linked to sport career termination, researchers have shown that some women continue sport careers once becoming mothers (Darroch & Hillsburg, 2017; McGannon et al., 2019). A closer look at these findings shows that elite athlete mothers face barriers and stressors. Some athletes experience guilt due to good mother ideals that emphasize caregiving over training, competition, and travel (Darroch & Hillsburg, 2017; Davenport et al., 2023). A sport career for athlete mothers is complicated by a lack of information about pregnancy or post-partum transitions back to sport (e.g., breastfeeding, training), poor funding and/or sponsorship, limited maternity policies and inadequate childcare (Darroch et al., 2019; Davenport et al., 2022). As women navigate sport careers during pregnancy and beyond, their stories are gaining media visibility. Researchers are studying media discourses to enhance understanding of motherhood and sport and the implications for sportswomen’s lives (e.g., McGannon et al., 2012; Scott et al., 2022). This research makes a valuable contribution to feminist sport media studies, whereby complex messages about sportswomen and gender (in)equality come to the fore (Bruce, 2016; Cooky & Antunovic, 2020).

The aim with our study is to build on the growing body of media research on athlete mothers. We accomplish this aim by using feminist narrative inquiry to interrogate the discrimination that two Canadian athletes (i.e., boxer Mandy Bujold and basketball player Kim Gaucher) faced on their journeys to the 2020 Tokyo Games. These athletes gained media attention when the International Olympic Committee’s (IOC) pandemic rules impacted their right to compete due to their motherhood status, and they pushed back. Bujold’s and Gaucher’s stories were also part of media conversations about Tokyo 2020 as the most ever gender-balanced Games juxtaposed with the IOC’s discrimination of women athletes (e.g., lack of safeguarding, sexist uniforms) (Pegoraro & Arndt, 2021). As Bujold’s and Gaucher’s stories brought attention to maternity rights, studying their journeys allows us to theorize visible feminism, gender (in)equality, and tensions for sportswomen, in media narratives (Cooky & Antunovic, 2020). To contextualize our study, we first discuss media research on Olympic athlete mothers, followed by a brief overview of neoliberal feminism. Next, we outline feminist narrative inquiry theory, purpose/research questions, research methods, and narrative analysis methodology. We then discuss three distinct narrative features (i.e., *motifs*) identified in our analysis feeding into two narrative threads. We conclude with reflections and future research.

## Literature Review

### Media Representations of Motherhood and Sport

The intertwining of motherhood and sport in the media is a longstanding issue that highlights tensions between the media’s recognition of sportswomen and stereotypical gendered portrayals (Bruce, 2016). Feminist sport media scholars’ syntheses of this research problematize media coverage of sportswomen as mothers and wives/girlfriends due to the media’s use of ‘ambivalent’ and ‘heteronormative’ frames (Bruce, 2016; Cooky et al., 2015). These media representation practices circulate narrow feminine ideals while trivializing women’s athletic accomplishments and reinforce sport as masculine (Bruce, 2016; Cooky et al., 2015). Despite these criticisms, as more women return to sport post-partum (Davenport et al., 2023), researchers are examining media representations of athlete mothers to gain insight into social constructions of motherhood and sport (McGannon et al., 2015).

Research on media representations of Olympic athlete mothers is relevant to our study of athlete mothers’ journeys to Tokyo 2020. A comeback narrative theme is prominent in previous research. Comeback narratives portray athlete mothers as navigating barriers linked to biology (e.g., weakened, or inferior bodies, natural desire to be mothers) and good mother ideals (e.g., prioritizing caregiving) to ‘come back’ to elite sport (McGannon et al., 2015). Researchers problematize comeback meanings by outlining ideologies of body surveillance, normative femininity, and individualism, which reproduce an ‘athlete mother’ trope fraught with tensions (McGannon et al., 2015). Cosh and Crabb’s (2012) discourse analysis of news media portrayals of Australian water polo player Keli Lane’s ambition to go to the 2000 Olympics, illustrates these themes. Lane became tabloid fodder due to carrying three pregnancies to term, allowing two children to be adopted. She was convicted of murdering the middle child at two days old. The trial was a media spectacle rendering Lane’s comeback quest to the Games as inconceivable, necessitating an extreme ‘athlete or mother’ dichotomy, with tragic consequences.

Additional media studies of Olympic athlete mothers highlight shifting representations of athleticism and motherhood, in comeback narratives. Hodler and Lucas-Carr (2016) interrogated news and sport magazine representations of US swimmer Dara Torres’ comeback to the 2008 Olympics. At 41 years of age, Torres was portrayed as a source of fitspiration who used training post-partum, to regain fitness and get her body back. These representations reproduce heterosexy sportswomen ideals, consumerism, and individualism. Torres’ comeback can also be read as producing a strong post-partum body achieving exceptional performance. McGannon et al.’s (2015) study on news media representations of team USA mothers in the 2012 Olympics, also shows portrayals of contradictory comebacks. The first comeback was one of physical hardship linked to women’s biological fragility, and the second comeback was one of physical and mental strength gained from motherhood. Both comeback meanings reinforce a good mother ideology that athletes uphold with minimal support, when ‘coming back’ to sport. Like Torres’ post-partum body rendered as achieving high performance goals, the second comeback resists prioritizing motherhood over sport, through a compatible elite athlete-mother identity. In contrast to these comeback themes, Dashper’s (2018) study of British heptathlete Jessica Ennis-Hill’s comeback in news coverage of the 2016 Olympics, identified the use of an ‘ambivalence’ frame. Ennis-Hill’s post-partum athleticism was constructed as incompatible with ‘elite’ performance, despite winning a silver medal. Ennis-Hill’s motherhood status was used to render her comeback as past her prime and downplay her athleticism.

Although media research findings on Olympic athlete mothers show ambivalent and heteronormative representations of comebacks, findings also draw attention to media representations disrupting stereotypical notions of motherhood and sport. To advance understanding, more research is needed engaging with what Bruce (2016) termed ‘new rules for new times’. A ‘new rules/new times’ focus moves beyond liberal feminist theorizing of ‘either or’ (e.g., mother or athlete, weak or strong, pretty, or powerful) in old frames (e.g., ambivalent, heteronormative). Contemporary feminisms (e.g., third wave, post-feminist, neoliberal) are used to account for the media’s renderings of sportswomen’s gendered subjectivity (Bruce, 2016).

One line of inquiry that holds potential to advance this understanding, is media stories about athlete mothers’ discrimination (e.g., lack of polices/support, being dropped by sponsors). When athlete mothers’ career tensions are centralized in the media, the analytical focus shifts from ‘comebacks’ to how gender (in)equality factors into motherhood and sport mediation. Engaging with feminist critiques of such stories affords the exploration of a media environment that portrays athlete mothers as powerful and autonomous, while inequalities persist in sport institution practices linked to motherhood status (Davenport et al., 2023). Although this line of inquiry is less studied in motherhood and sport research, one exception underscores the value of focusing on discrimination stories for theorizing ‘new sporting femininities’ (Toffoletti & Thorpe, 2018). Scott et al.’s (2022) feminist framing analysis explored news articles and tensions for US Olympic runner parents (e.g., Alysia Montaño and Allyson Felix) related to corporate sponsors. Findings included framings of exploitation versus empowerment whereby sponsors recognized mothers’ market value yet cut contracts. Frames of reactivity versus proactivity also showed sponsors framed themselves as supportive, while athletes noted support came from their own proactive advocacy. This feminist analysis shows the cultural change needed in corporate spaces to support athlete parents beyond exploitive, reactive, and performative practices.

### Neoliberal Feminism: Contextualizing Contemporary Motherhood

To contextualize our narrative analysis, neoliberal feminism warrants discussion. Neoliberal feminism is a form of feminism in culture and media texts, emphasizing neoliberal values of individualism, entrepreneurialism, and economic opportunity, as solutions to gender inequality (Banet-Weiser et al., 2020). Feminist media scholar Angela McRobbie (2020) emphasizes a coupling of liberal feminism with neoliberal feminism, with gender inequality acknowledged, but often without change or with less disruption of the status quo. This lack of disruption results in equity solutions and social change being framed by neoliberal ideologies of feminist self-governance and independence, in a for-profit market economy (McRobbie, 2020).

An upwardly mobile feminist subject who engages in reproductive and care work is at the centre of contemporary neoliberal feminist conversations (Banet-Weiser et al., 2020). In turn, maternal and labour empowerment are feminist ideals, circulated in media representation practices rendering motherhood as ‘self-regulated’, and ‘balanced’ with (un)paid work (McRobbie, 2020). For women who want to become mothers, neoliberal practices linked to body self-governance (e.g., family planning) and career investments (e.g., time and sacrifice) are part of ‘good motherhood’ in neoliberal feminism (Rottenberg, 2014). Working mothers can achieve success in separate spheres of domestic and work-life, through an illusion of balance, individualism, and intensive mothering ideologies, while social and structural support are disavowed (McRobbie, 2020). Incorporating a neoliberal feminist critique into the mediation of motherhood and sport, holds value to extend understanding of maternal media stories, in narratives. As noted in our literature review, sportswomen now navigate careers in a media environment that portrays athlete mothers as powerful, athletic, and autonomous, while cultural and structural barriers persist. We will explore these tensions linked to neoliberal feminism in media stories of athlete mothers’ discrimination, using feminist narrative inquiry.

### Theoretical Framework: Feminist Narrative Inquiry

To bring a feminist analysis of athlete mother discrimination media stories to the fore, we grounded our study in relativist feminist narrative inquiry. Narrative inquiry is part of the narrative turn in the social sciences centralizing stories as cultural sites of identity formation, meanings, and (in)action (Woodiwiss et al., 2017). A key tenet of relativist narrative inquiry is that identity-related stories and (in)actions connected depend on cultural narratives available (Smith & Sparkes, 2009). Although the term ‘narrative’ is often interchanged with a ‘story’, narrative scholars distinguish between the two for theoretical and analytical purposes (Smith & Sparkes, 2009). A ‘story’ is a tale that people tell about themselves or others, containing (auto)biographical information brought to life by centralizing events and happenings (Smith & Sparkes, 2009). A ‘narrative’ is a cultural resource that people draw on to tell stories–including media stories–with patterns of meaning and outcomes (Smith, 2021a). Narrative researchers are interested in how certain elements (e.g., characters, events/happenings, recurring concepts) (re)create contextual meaning in stories, in narrative threads (Smith, 2021a). Given these assumptions, researchers interested in socially constructed identities, meanings, and actions, give analytical attention to *stories* and *narratives* that frame them (Smith & Sparkes, 2009).

Feminist narrative inquiry centralizes the study of women’s stories as constraining and/or emancipative, depending on social, ideological, and structural forces (Woodiwiss et al., 2017). While other feminist perspectives (e.g., post-structuralism, framing theory) have a similar focus when studying ‘media narratives’ (e.g., Hodler & Lucas-Carr, 2016; Scott et al., 2022), feminist narrative inquiry gives analytical attention to thematic content of women’s *stories*, framed by *cultural narratives* (Woodiwiss et al., 2017). In this sense, feminist narrative inquiry scholars take a ‘story analyst’ approach to stories as ‘objects of study’, producing an analytical account of narrative features that (re)create stories (Smith, 2021a). In feminist narrative inquiry, athlete mother identities are theorized as socially constructed as they interpret the world using *narrative practices* (e.g., tell stories of struggle and/or empowerment) that allocate certain meanings (e.g., athletes navigate motherhood with(out) support), tied to ideologies (e.g., neoliberalism) circulated in media stories (McGannon et al., 2019). The intent with identifying building blocks of stories and ideologies in narratives (Smith, 2021a), is to uncover what is silenced and taken for granted about women’s lives (Woodiwiss et al., 2017). Using feminist narrative inquiry as a theoretical lens in conjunction with neoliberal feminism, affords a novel exploration of athlete mother discrimination stories concerning contradictions and power, in media narratives. Feminist narrative inquiry complements feminist media scholars’ calls to expand feminist theorizing of gendered subjectivities (Bruce, 2016), extended to the mediation of motherhood and sport.

### Purpose and Research Questions

Although much has been learned by studying distinct media narratives surrounding athlete mothers, more research is needed to understand the tensions that thread stories, particularly in terms of discrimination meanings. Given the potential of this line of inquiry to advance the study of sportswomen’s gendered subjectivity using contemporary feminisms (Bruce, 2016; Cooky & Antunovic, 2020; Toffoletti & Thorpe, 2018), our study aims to build on research centring media stories of athlete mother discrimination. We accomplish this aim by using feminist narrative inquiry to interrogate and theorize, two Canadian athlete mothers’ mediated journeys to the 2020 Olympics, as the product of socially constructed meanings circulated in stories and narrative threads. The following research questions guided the study: 1. what stories and narratives are drawn on in sport media to create discrimination meanings and gender (in)equality? and 2. what are the ideological and social implications of discrimination and gender (in)equality meanings identified?

## Methods

### Canadian Athlete Mothers and Key Media Stories

In the four months preceding the Tokyo Games, Bujold and Gaucher each had one child. Bujold’s daughter Kate was born on November 5, 2018, and Gaucher’s daughter Sophie was born on March 19, 2021. Both athletes planned their pregnancies at the height of their sport careers, with no plans to retire post-partum. Bujold and Gaucher were touted as Canada’s hopefuls for medals in Tokyo 2020, due to excelling in their sports. Career highlights for 33-year-old Bujold included 11 national titles, gold medals in the 2011 and 2015 Pan American Games and a 5^th^ place finish at the 2016 Rio Games. At 37 years of age, Gaucher was a veteran on the 4^th^ ranked Canadian basketball team heading into Tokyo. Her accomplishments included a gold medal in the 2015 Pan American Games and playing in the 2012 and 2016 Olympics.

To answer the research questions, we collected mainstream media stories surrounding the discrimination athletes faced linked to their motherhood status and IOC’s rules. In Bujold’s case, because the competitive year prior to the Games was canceled due to the Covid-19 pandemic, the IOC ruled that boxers from the Americas would qualify for Tokyo based on three tournament rankings from 2018 and 2019 (Heroux, 2021). Despite a 2^nd^ place ranking in the Americas pre-pregnancy and three top finishes post-partum, the qualifying tournaments excluded Bujold for an Olympic spot due to being pregnant in 2018 and early postpartum in 2019 (Heroux, 2021). After the IOC denied Bujold’s request to consider her pre-pregnancy ranking, she appealed to the Court of Arbitration in Sport. Gaucher was negotiating a sport career and new motherhood four months before the Games, which included breastfeeding her 3-month-old daughter. These tensions were compounded by Games organizers excluding family and fans to enter the country as part of Covid-19 protocols (Grange, 2021b). After Gaucher’s request for breastfeeding athletes to bring their families was denied, on June 23 she posted five videos on Instagram. The posts gained mainstream media attention, elevating the plea to bring her daughter to the Games. At the end of June, a resolution came for both athletes when Bujold won her appeal, and an exception was made for breastfeeding athletes’ families to attend the Games.

### Data Collection

The analysis of Bujold’s and Gaucher’s stories is the result of considering the ‘suitability’ and ‘feasibility’ of this qualitative study (see Tracy, 2020). Tracy (2020) suggests that ‘suitable’ research projects should “encompass most, if not all of the theoretical issues and characteristics of interest in terms of the research topic or problem” (p. 12). Toward that end, we were interested in the contentious issue of contemporary media representations of athlete mothers, against a backdrop of an inequitable sport system. As researchers of gender equity and sport, we were also aware of how the pandemic disadvantaged sportswomen, particularly athlete mothers. Our literature review further identified a need for research on athlete mother discrimination stories, and that a feminist narrative inquiry lens along with neoliberal feminism, would advance feminist theorizing of sport media. Based on these points, Bujold’s and Gaucher’s stories were deemed ‘suitable’ to answer the research questions and build theoretical knowledge, due to the following characteristics: 1. both were discriminated against due to motherhood status, 2. there was rich extensive mediation on their discrimination, and 3. discrimination stories covered their Olympic journeys during the pandemic. All these points culminated into a ‘feasible’ study, as we gathered mainstream media stories readily availablefrom North American sport media sources, to explore this timely topic using feminist narrative inquiry.

Data collection was delimited to English language stories in Google News database, using each athlete’s name plus search terms ‘Tokyo Olympics’ and ‘motherhood’. Time frames included stories appearing 3.5 months before the Games (i.e., April 1, 2021-July 22, 2021), during the Games (i.e., July 23, 2021–August 11, 2021), and one-month post-Games (i.e., August 12-September 30, 2021). These time frames afforded exploration of each athlete’s journey as a mother leading up to, during, and after, the Games. Search terms for Bujold yielded 110 stories and search terms for Gaucher yielded 78 stories from North American news and sport media in digital/online formats. To yield in-depth stories about athlete mother discrimination, stories were read by the first three co-authors, resulting in removal of duplicates, stories mentioning the athletes in passing, and stories that did not discuss motherhood. We retained 103 detailed stories (i.e., *n* = 48 for Bujold, *n* = 30 for Gaucher, *n* = 25 for both athletes), with most stories (*n* = 93) coming from Canadian outlets (e.g., CBC sports, Sportsnet) and ten from US outlets (e.g., USA Today). These outlets have a national presence, although a small number of regional Canadian outlets (*n* = 6) also covered the athletes. None of the articles retained were identified as feminist sport media outlets or only covered women’s sport.

### Thematic Narrative Analysis: Identifying Plots and Narratives

Media stories were subjected to a social constructionist thematic narrative analysis using principles from Smith, 2021a) to identify content in stories that shape discrimination meanings in narrative threads. To analyze story content, we identified *motifs* across the corpus of media stories*. Motifs* are recurring aspects of stories (e.g., events/happenings, conflict/tensions, action, certain phrases) that create a web of meanings, shaping narrative themes (see Saldaña, 2021). This analytic approach aligns with relativist feminist narrative inquiry by viewing media stories as social constructions, centralizing stories, linked to ideologies and narratives impacting women’s lives (Woodiwiss et al., 2017). We achieved meaningful coherence by aligning methods with research aims, connecting analysis with research questions, interpretations, and narrative theory (Smith, 2021a). Following data organization, the first three co-authors engaged in ‘narrative indwelling’ by (re)reading stories, and recording impressions related to research questions (Smith, 2021a). Indwelling commenced separately, followed by co-authors meeting as critical friends, to discuss impressions (Smith & McGannon, 2018). The research team initially oriented around passages for Bujold related to being ‘deserving to compete’ due to athletic status, and family planning aligned with an athlete-mother identity. Passages in stories for Gaucher also related to her being ‘deserving to compete’ due to veteran status and family planning. As discussion proceeded, we noted that story passages centred on both athletes’ rights as rescinded by the IOC’s gender inequality, forcing them into an athlete *or* mother identity.

The principle of ‘identification and refinement of narrative patterns and relationships’ was facilitated by the first author’s closer readings of story passages (Smith, 2021a). This principle was a recursive process of writing summaries linked to story excerpts for both athletes, and (re)shaping meanings related to struggles, and resistance, linked to motherhood and sport. Questions such as “what are common thread(s) about motherhood and sport?” and “what thread(s) occur consistently or offer resistance?” facilitated this process. One candidate *motif* was ‘entitlement to compete’ intertwined with an ‘IOC hypocrisy’ narrative thread. These were refined into a ‘forced to choose’ *motif* encompassing tensions between athlete *and/or* a mother and an *exposing a motherhood penalty* narrative to link neoliberal ideals with discrimination. Another candidate *motif* in resolution stories was ‘athlete activism’. This *motif* was refined into ‘more than us: a great day for women’s sport’ to show conformity and collective resistance tensions linked to discrimination meanings in stories. The final principle involved ‘thinking with narrative theory’ by using literature to interpret story *motifs* and ideologies forming meanings by noting similarities and differences, and *motif* content in media stories (Smith, 2021a). This principle is reflected in a combined findings and discussion section to balance description and interpretation of the data, and methodological coherence of our study (Smith, 2021a).

## Findings and Discussion

We identified three *motifs* in media stories: *last shots*, *forced to choose*, and *more than us*. The *last shots* and *forced to choose motifs* intertwined to expose nuanced discrimination meanings, feeding into a narrative thread of *exposing* a *motherhood penalty*. The *more than us motif* featured primarily in stories covering the resolution for both athletes to compete in the Games, culminating into *a good day for women’s sport* narrative thread.

### Last Shots and Forced to Choose: Exposing a Motherhood Penalty

In the *last shots motif*, Gaucher and Bujold were portrayed as veterans for whom the Tokyo Games would be the last chance to compete, due to impending retirements. This *motif* shows conflict through outlining tensions both athletes experienced, when the ‘last shot in their careers’ turned into a last hope as the IOC made no considerations for athlete mothers*.* Tensions were created in this *motif* by journalists outlining sacrifices both athletes made through family planning and hard work to regain athletic status, only to be thwarted and denied competing:

“That's a very real possibility, that I could go back, get back in shape, feel great [and decide not to go to Tokyo],” says Gaucher…It wasn’t the plan. Gaucher had previously tried to time pregnancies for off-years in the Olympic cycle and the rest, but – quite literally – life happened. “Turns out, you can’t plan these things,” she says. (Grange, 2021a, paras. 20–23).

The 11-time Canadian flyweight champion has spent money, time and energy getting herself back in contention only to find out — about three weeks before the qualifier no less — that her shot at making the Games is gone for reasons out of her control. It’s not her fault the qualifier was cancelled. It’s not her fault the pandemic essentially wiped out the last year of competition. And she definitely shouldn’t be punished for missing the events that count toward the selection rankings because she was starting a family (Brown, 2021, paras. 10–13).

The above excerpts show discrimination meanings identified in previous studies that include timing pregnancies, investment of time and money to return to sport, and navigating physical realities (Bennett et al., 2022; Davenport et al., 2023). These meanings culminate into stress linked to precarious timing of competition cycles, due to less maternity and post-partum support from sport governing bodies (Davenport et al., 2022; Scott et al., 2022). The features of the *last shots motif* and *motherhood penalty* narrative thread can be theorized as linked to neoliberal ideals identified by feminist scholars (Banet-Weiser et al., 2020). In neoliberal feminism, scholars problematize how mothers govern themselves to make empowered choices (e.g., family planning, career), while government and structural support for family is withdrawn (McRobbie, 2020). Journalists problematized these individualized solutions for Gaucher and Bujold, as they become what McRobbie (2020) calls ‘entrepreneurial subjects’ in a sport marketplace doing little to address, and perpetuating, inequality for athlete mothers.

The *last shots motif* further exposes an emotional labour penalty, identified in research on Olympic parents, amplified by the pandemic due to career uncertainty and small competition windows due to fertility age (Bennett et al., 2022). Our narrative analysis of Bujold’s and Gaucher’s journeys also shows that the notion of *time* was used to shape a *last hope tension* in the *last shots motif*. A *last hope tension* is an additional emotional penalty incurred by Gaucher and Bujold, due to neoliberal economic control the IOC maintains over athletes. These points are shown in stories highlighting how both athletes’ requests remained contentious and unresolved two weeks before the Games. As one story reported, “Gaucher called it time for “a hail mary” in a series of Instagram videos explaining the situation” (Negley, 2021, par. 3). Gaucher’s last hope exposed emotional aspects of a neoliberal motherhood penalty, as she devised her own solutions (e.g., pumping milk 28 days in advance). As time ticked down for Bujold, the *last shots motif* also exposed a *motherhood penalty* narrative linked to an emotional labour penalty:

Ms. Bujold, meanwhile, admits the arbitration process is a distraction at a time when she would rather focus on training for an event that she had long planned to be the bookend of her remarkable boxing career. “It can be hard to focus with this huge uncertainty hanging over you. There’s definitely moments where you’re like ‘What’s the point? Why am I doing this?’” she said (Mercer, 2021, paras. 16–17).

Incorporating Gaucher’s and Bujold’s voices into the *last shots motif* shows the emotional fall out from discrimination exposed in media stories, which further shows the media’s centring of ‘feminist consciousness’ identified by feminist media scholars (McRobbie, 2020).

An additional *forced to choose motif* identified in our analysis further exposes the injustice a *last shots* situation created by the IOC acting as arbiter over women’s lives in a neoliberal market economy. The following story excerpt again shows the integration of Bujold’s voice to expose the IOC’s rules as outdated, within the context of the media’s centring neoliberal feminist push back on *forcing women to choose* between being an athlete *or* a mother:

The boxer says the rules that disqualified her are a holdover from an era when many Olympic-calibre athletes would quit their sport when they wanted to become a mother. Ms. Bujold argues the IOC doesn’t seem to think qualification periods should be flexible to accommodate women during their pregnancies. “I don’t think athletes should have to wait for their career to be done to start a family,” she said. “I think this is just ancient thinking. We have to move into this century, and say ‘This is happening more and more, so how can we protect these athletes so they can take that time to have a child?’” (Mercer, 2021, para. 9–10).

Neoliberalism further demands career dedication with, or without, support and a neoliberal feminist project of work-life balance for mothers is supposed to reconcile these tensions (McRobbie, 2020). As a breastfeeding mother 3.5 months post-partum, Gaucher was represented in stories as *forced to choose* between competing or staying with her baby, as she tried to ‘balance’ being an athlete and a mother, to compete without concessions. Stories frequently quoted Gaucher’s Instagram post where she said, “…right now I’m being forced to decide between being a breastfeeding mom or an Olympic athlete. I can’t have them both” (Negley, 2021, para. 4).The *forced to choose motif* further exposes a motherhood penalty of a lose-lose situation. Neoliberal feminist tensions of choice are compounded in the pursuit of ‘balancing’ domestic and work spheres outlined by feminist media scholars (McRobbie, 2020). These points are shown in the following story excerpt about Gaucher, intertwined with an emotional penalty linked to being *forced to choose* between her career or mothering practices:

It leaves Gaucher facing a ‘lose-lose’ choice: if she decides to finish off her decorated basketball career by trying to win a medal – a seemingly impossible dream when she first started with the program when even qualifying for the Olympics was a mountain too high – it would mean being away from her newborn daughter for more than a month. If she decides that she and Sophie can’t bear that kind of separation at this stage, it will mean missing out on a goal that has been nearly two decades in the making (Grange, 2021a, paras.19–20).

Bujold’s choice was problematized in media stories as *forced* on her by the IOC despite her (so-called) neoliberal choice to time pregnancy and resume a career promised to women, outlined in neoliberal feminist theorizing (McRobbie, 2020). In the *forced to choose motif*, the fate to compete lay in the hands of the Court of Arbitration for Sport, as Bujold fought for maternity rights. A neoliberal feminist ideology intertwined with tensions in the *forced to choose motif* is shown in a story excerpt exposing the IOC’s sexist rules compromising Bujold’s choice:

She gave birth to her daughter, Kate Olympia– or K.O.– and stepped back into the ring in 2019, planning a comeback in Tokyo. Then the COVID-19 pandemic hit, cancelling all qualifying events…The IOC’s task force ruled Ms. Bujold, who’s won 11 Canadian championships, two Pan American Games titles, and was a medal favourite at the Rio de Janeiro Games in 2016, did not have enough points to qualify for Tokyo. Whether Ms. Bujold is allowed to compete in Tokyo will now be decided outside the boxing ring – by the Court of Arbitration for Sport, based in Lausanne, Switzerland (Mercer, 2021, paras. 4–6).

Although stories invoked the IOC to outline Gaucher’s and Bujold’s situations and *forced choices* due to extenuating pandemic circumstances (e.g., Karstens-Smith, 2021), the *last shots* and *forced to choose motifs* also drew on a ‘cast of characters’ (Smith, 2021a) to elevate the IOC as sexist and discriminatory. Incorporating social agents into stories show contradictions concerning the *exposure of a motherhood penalty* narrative that simultaneously critiqued sexism, while embracing neoliberal feminist ideals, in stories. High profile women athletes (e.g., Billy Jean King, former Canadian hockey player Hayley Wickenheiser) and Canadian government officials were also invoked in stories to elevate Bujold’s and Gaucher’s cases as human rights violations. In some stories, ‘characters’ were quoted together, shown in the following excerpt outlining the Sport Canada Heritage Minister’s letter to the IOC juxtaposed with former boxer Lennox Lewis’ social media post:

I urge you to reconsider the decision to exclude Mandy Bujold, in the name of fairness and on the basis of our mutually stated ambitions of gender equity, human rights and promoting women and girls participating in sport,” Mr. Guilbeault wrote in a recent letter. Lennox Lewis, one of Canada’s greatest boxers, also slammed the IOC’s decision. “I find it preposterous that the Olympics in effect has denied her the opportunity to once again represent Canada,” he said on Twitter. “It’s especially confounding, when I’ve heard that the Olympics have made exceptions for other women who have also made the same choices to start a family and still pursue Olympic dreams.” (Mercer, 2021, paras. 12–13).

The above quote shows the media’s portrayal of high profile Canadian characters commitment to gender equity. The quote can also be read as performative and reactive, as the ‘mutual interest’ and ‘pursuit of dreams’ is centralized in the neoliberal economic enterprise of the Olympics. Moreover, feminist neoliberal values are upheld, as there is verbal support for a ‘working athlete mother’, but no call for structural change. In contrast to the above excerpt, Bujold’s lawyer Sylvie Rodrigue was also brought into some stories to expose the IOC’s violation of human rights, but shows her commitment beyond performative gestures:

In the Olympic Boxing Task Force’s revised ranking system for Tokyo, “it’s like Mandy has never been ranked in the world,” Rodrigue said. “What we say is the fact that they do not accommodate pregnant or postpartum athletes by recognizing their rankings pre-pregnancy, they are violating the rights of the athletes from a gender equity and from a discrimination standpoint,” she said (Toronto AP, 2021, paras. 7–8).

The ways in which these characters were featured in the *forced to choose motif* to critique sexism, shows that ‘feminist resistance’ went mainstream through collective voices shown in feminist media research on female athlete activism (Cooky & Antunovic, 2020). We explore these points and tensions in greater detail in the next *motif* and narrative thematic thread.

### More than Us: A Good Day for Women’s Sport

The *more than us motif* shows that both athletes’ exposure of *a motherhood penalty narrative* in the media*,* coalesced into something bigger: fighting for women’s rights, and inspiring future generations of women and girls, including their own daughters:

“When I started this whole thing, when we said I wanted to come back for the Olympics, I wanted to be able to inspire, not just future young Canadian basketball players, but my daughter Sophie, to be able to tell her that I gave it my all,” Gaucher said (Smith, 2021b, para.19).

“Years down the road, I'm going to have a conversation with my daughter about this stage in my boxing career, I'm now going to be able to tell her that I took time off to become a mom and came back a stronger, better woman and proved that you can have a family and be an Olympian,” Bujold said. “This decision has impacted not only my future, but also the future generation of young girls.” (Canadian Press, 2021, para. 3).

The above quotes show feminist consciousness in stories using this recurring *motif*, through demanding equity for others, which can be read as a form of ‘collective activism’ shown in previous feminist media studies of female athletes (Cooky & Antunovic, 2020). Gaucher’s and Bujold’s inspirational journeys in the *more than us motif* also make a neoliberal feminist move of positioning adversity (i.e., the fight) and self-determination as a pathway for women to follow, rather than push for structural change called for by feminist media scholars (McRobbie, 2020). These points are further accomplished by a *good day for women’s sport* narrative, as the *more than us motif* intertwined with a neoliberal ideology to articulate a ‘feminist victory’ (CBC Sports, 2021; Karstens-Smith, 2021). This victory was declared in stories featuring both athletes who won ‘battles before the Games began’ (CBC Sports, 2021; Smith, 2021b), and calling this a ‘MOMumental day’ (Campigotto, 2021) for women’s sport, due to the resolutions to compete:

“It is a great ending,” Bujold said. “We fought hard for it, we did everything within our control, we literally left it on the ring, it's probably the easiest way to say it.” The news came on a momentous day for Canadian female athletes. Earlier Wednesday, the IOC announced that breastfeeding athletes can have their babies with them in Tokyo, a move that comes a week after Canadian basketball player Kim Gaucher made an emotional plea to bring three-month-old daughter Sophie to the Games. (Canadian Press, 2021, paras 12–13).

Gaucher and Bujold were also characterized as ‘trail blazers' and ‘activists' in the *more than us motif* as the media validated their feminist push for maternity rights, which led to landmark change. These findings are akin to Cooky and Antonuvic’s, (2020) feminist media analysis findings of the USNWT’s fight for equal pay lawsuit, which revealed the media’s support for gender equity. Stories about Gaucher’s and Bujold’s *more than us* fight drew on a renewed feminist consciousness, characteristic of neoliberal feminism identified by feminist media scholars (Banet-Weiser et al., 2020). Gaucher and Bujold were often portrayed together in stories in the *more than us motif*, which reinforced their identities as ‘trail blazers’ for maternity rights. A story by Dichter (2021) illustrates how both athletes’ feminist push back led to a renewed feminist fight to compete, linked to gender equality along with a neoliberal ‘Olympic dream’:

“To all of the working moms out there who have had to fight this fight before, I think it was just a really good day for women in sport today,” Gaucher said on Wednesday, one day after she was officially named to Canada's Olympic basketball roster. Bujold said her fight was the most difficult of her career. “In 2021 I didn't ever think I would have to fight this battle. I can say that this has been one of the biggest fights of my career, but also the fight with the most meaning. I was standing up for what I believe is right and for the dream that I had worked so hard for,” Bujold said (Dichter, 2021, paras. 4–6).

Media stories using *the more than us motif* also drew on neoliberal feminist ideals of empowerment fused with a compatible ‘athlete-mother’ identity, identified in media research on Olympic mothers (McGannon et al., 2015). The importance of a compatible identity to afford athlete mothers training and competing with less guilt and neoliberal work-life ‘balance’, is shown in previous research (Darroch & Hillsburg, 2017; McGannon et al., 2019). However, without social and policy support, researchers also expose that athlete mothers are left on their own to overcome barriers (Davenport et al., 2022; Scott et al., 2022). A story in which Bujold discussed her journey and resolution, illustrates these tensions:

I want to send a strong message to all the female athletes who have put their personal goals and dreams aside because they are afraid of the consequences: You don't have to pick one or the other. You can be a mom and perform at the highest level. You can be a wife or partner and win championships or medals. You can be a friend and break world records or hit personal bests. You have the ability to view obstacles not as barriers, but as opportunities to overcome. Go over them. Go around them. Go right through them if you need to. But never for one moment feel as though you're limited because you're a woman (Bujold, 2021, para. 18).

The above example shows the tensions between neoliberal feminist ideals and landmark change concerning maternity rights, as the media centres Bujold advocating for career accomplishments (e.g., winning championships/medals) intertwined with a ‘feminist subject’ (i.e., mother) pushing back on the system. Solutions offered are what McRobbie termed ‘feminist resilience’ (2020), whereby obstacles are not removed, but shifted through women’s mindset (e.g., “view obstacles not as barriers but as opportunities to overcome”). Feminist scholars have problematized such pathways to change as centring White, heterosexual, cis-gendered women in a palatable form of inspirational feminism, whilst maintaining capitalist and patriarchal power structures (McRobbie, 2020). We explore these notions further in our conclusions section.

## Conclusions

In this study, we expanded feminist theorizing in the sport media complex by using ‘new rules for new times’ (Bruce, 2016), through focusing on two athlete mothers’ discrimination stories (i.e., Mandy Bujold and Kim Gaucher), using feminist narrative inquiry. We also incorporated a neoliberal feminist critique into our interrogation of the mediation of Bujold’s and Gaucher’s discrimination on their journeys to the Tokyo Games. By using these feminist theories, we added to, and expanded theorizing ‘new sporting femininities’ (Toffoletti & Thorpe, 2018), in a changing media landscape. As such, our study’s findings move beyond liberal feminist critiques of media representations of athlete mothers as circulating narrow feminine ideals and trivializing women’s athletic accomplishments (see Cooky et al., 2015; Dashper, 2018). The tensions we identified through the intersecting *narrative motifs* of l*ast shots, forced to choose*, and *more than us*, also builds on media research centralizing Olympic athlete mothers’ comebacks (Cosh & Crabb, 2012; Hodler & Lucas-Carr, 2016; McGannon et al., 2015) and discrimination stories (Scott et al., 2022). In what follows, we outline some key theoretical contributions from our study, in relation to our findings. We close this section with some practical considerations related to findings within the context of feminist narrative inquiry.

The intersecting *last shots* and *forced to choose motifs* identified in our thematic narrative analysis allowed us to expose the impact of a motherhood penalty on the lives of two athlete mothers who are feminist subjects engaged in neoliberal family planning, hard work, and balance, without support (McRobbie, 2020). Despite previous media studies identifying Olympic athlete mothers portrayed as autonomous, powerful, and making ‘choices’ affording ‘come backs’ (see Hodler & Lucas-Carr, 2016; McGannon et al., 2015), our neoliberal feminist critique of Bujold’s and Gaucher’s stories shows the futility of individualized choices for athlete mothers. Their stories in a motherhood penalty narrative thread showcase the lack of concessions for maternity rights by the IOC and Games organizing committee which *forced these athletes to choose* and placed them in a *lose-lose situation*. In turn, Bujold and Gaucher, were subjected to undue stress prior to the Games and compounded gender inequity for sportswomen arising from the pandemic (Pape, & McLachlan, 2020). Moreover, some stories noted that despite the resolution to compete and IOC concessions, Bujold still awaited a seeding spot in Tokyo (CBC Sports, 2021). The decision came far too late for other breastfeeding athletes to make plans to bring their families to the Games (Karstens-Smith, 2021). When theorized using neoliberal feminism, the tensions we identified allow for the exposure of persistent inequalities in sport institution practices (Davenport et al., 2022), bringing a pressing need for more socio-cultural change concerning maternity rights .

Our findings concerning maternity rights underscore a marked shift in media representations of the ‘athlete mother’ trope as a dichotomy of ‘unable to compete’ versus a ‘super mum’ (McGannon et al., 2015), to one that elevates athlete mothers’ feminist consciousness and voices to demand an equitable right to compete. Toward that end, our narrative analysis further reveals that Gaucher’s and Bujold’s’ collective stories of fighting for their rights and rights of other women, led to landmark change in maternity rights on an international level. These findings show that in neoliberal feminism, structural change happens when women collectively push-back and are supported by the media (Cooky & Antunovic, 2020; Scott et al., 2022). Our analysis also highlights tensions intertwined with this international landmark change, as *the more than us narrative motif* linked with a *good day for women’s sport* happy ending partly eclipses ongoing realities for athlete mothers. Despite maternity rights in sport changing in some spaces (e.g., corporate sponsors, see Scott et al., 2022), additional reform is needed to dismantle the patriarchal structures penalizing athlete mothers, problematized by neoliberal feminist scholars’ critiques (McRobbie, 2020; Rottenberg, 2014). Notably, in Bujold’s and Gaucher’s home nation of Canada less than 1/3 of national sport organizations have maternity policies (Davenport et al., 2022). Additionally, no policies exist in the Canadian sport system explicitly addressing motherhood penalty discrimination meanings identified in Gaucher’s and Bujold’s stories, although researchers have suggested changes (Davenport et al., 2022; 2023).

The *more than us motif* threading Bujold’s and Gaucher’s stories linked with neoliberal inspiration, resilience, and ‘trailblazer' identities, can also be problematized as limiting athlete mother activism portrayals in sport media (Scott et al., 2022). In the motherhood activist movement, a neoliberal feminist project of aspirational goals and individual solutions to barriers are criticized by feminist researchers as centring heterosexual White women’s rights (Banet-Weiser et al., 2020). These points are underscored by sport media researchers problematizing Black women’s activism as downplayed or ignored, often eclipsed by men’s activism and/or neoliberal feminism serving White women’s interests (Brown, 2018; Cooky & Antunovic, 2020). Black women exposed athlete mothers’ struggles well before Gaucher’s and Bujold’s Olympic journeys. In addition to Alysia Montaño and Allyson Felix exposing sponsorship discrimination outlined in previous media research (Scott et al., 2022), Felix and Serena Williams brought Black women’s pregnancy and health risks to light. Williams also led the change in tennis to retain rankings post-partum, and WNBA players have led pro-contract changes in maternity rights (Tsui, 2019). Gaucher’s and Bujold’s win for maternity rights was rightfully elevated by the media through the *last shots, forced to choose and more than us motifs*. These recurring *narrative motifs* centralized sportswomen’s rights without deferring to men’s sport as the default (Bruce, 2016; Cooky & Antunovic, 2020). However, the stories also missed an opportunity to elevate Black women’s leadership in maternity rights activism and draw attention to inequity faced by racialized athlete mothers (Scott et al., 2022). Feminist media scholars’ critique of neoliberal feminism’s aspirational White women can ‘do and be anything’ persona, also shows this trope risks upholding systemic barriers in maternity rights (McRobbie, 2020).

In relativist narrative inquiry, the central theoretical tenet that ‘stories do things’, points to the applied value of media stories as entry points for understanding and change (Smith, & Sparkes, 2009). Stories – in this case athlete mothers’ discrimination stories centralized in the media- have potential for (re)action as they allow for witness and reflection (Woodiwiss et al., 2017). Our narrative analysis of Bujold’s and Gaucher’s discrimination stories not only exposed a motherhood penalty (e.g., emotional labour, hard work, career setbacks), but our findings afford an opportunity for expanded conversations and solutions, concerning maternity reform. Although our study was limited to North American media stories available through Google News, our findings suggest that conversations should centralize recovery of women’s sport post-Covid-19 (Pape & McLachlan, 2020), and include athlete parental rights on the agenda. Athlete mothers are not merely ‘super women’ coming back to sport and high performance careers (Davenport et al., 2023) or ‘inspirational trailblazers’ fighting the system. They also face disproportionate mental health, career, and socio-economic penalties (Bennett et al., 2022; Scott et al., 2022). Our findings offer an opportunity to further engage with policy beyond neoliberal performative or reactive solutions (Scott et al., 2022). Policies might include extended paternity (not just maternity) leave, adjusted qualifying period/modified rules for athletes on paternity leave, paid childcare and family travel plus accommodations (e.g., Olympic village for families). Provision of sport science staff who support physical (e.g., breastfeeding, post-partum training) and psychological tensions/adaptations through mental health services (Davenport et al., 2023) would also be of value.

### Future Research Directions

Our research focus on the mediation of Canadian athlete mothers’ discrimination meanings in their journeys to Tokyo 2020 points to consideration of future research directions. An intersectional feminist lens that exposes systemic and structural contributors that penalize athlete mothers needs exploration, and centring, in media conversations of athlete parental rights (Scott et al., 2022). This focus would move beyond feminism in media narratives that benefits a select few. Research accounting for, and theorizing, intersectional athlete mother identities (e.g., race, sexual orientation, disability) circulated in media stories would contribute to these goals. A limitation of our study was the focus on media representations of White heterosexual cis-gender Canadian athletes. Exploring racialized athlete mothers of different sexual orientations from non-Westernized nations, would advance gender equity and parenting in sport media research. A transnational feminist lens to consider the international reach of stories and narratives concerning feminist consciousness and maternity reforms for sportswomen, would also be useful.

As our study, and previous research on elite athlete mothers, explores mainstream media outlets with a national presence, it would be useful to expand investigations by studying feminist sport media outlets centring these sportswomen’s stories. Further research is needed on media, sport and motherhood using an intersectional lens, across different sports, and different career stages, to explore nuanced transitions and implications. There is also minimal research on social media, sport and motherhood using feminist narrative inquiry or other feminist theorizing. Studying social media forms (e.g., Facebook, Twitter, Instagram) in relation to networked (self)representations of athlete mothers, would build on feminist sport media work that accommodates multiple entangled feminisms to explore contemporary gendered renderings of, and consequences for, sportswomen (Cooky & Antunovic, 2020; Toffoletti & Thorpe, 2018).
